# The evolution of molluscs

**DOI:** 10.1111/brv.12439

**Published:** 2018-06-21

**Authors:** Andreas Wanninger, Tim Wollesen

**Affiliations:** ^1^ Department of Integrative Zoology University of Vienna Althanstrasse 14, 1090 Vienna Austria

**Keywords:** Bilateria, BMP, body axis, Cambrian, development, EvoDevo, Hox, Lophotrochozoa, Mollusca, morphology

## Abstract

Molluscs are extremely diverse invertebrate animals with a rich fossil record, highly divergent life cycles, and considerable economical and ecological importance. Key representatives include worm‐like aplacophorans, armoured groups (e.g. polyplacophorans, gastropods, bivalves) and the highly complex cephalopods. Molluscan origins and evolution of their different phenotypes have largely remained unresolved, but significant progress has been made over recent years. Phylogenomic studies revealed a dichotomy of the phylum, resulting in Aculifera (shell‐less aplacophorans and multi‐shelled polyplacophorans) and Conchifera (all other, primarily uni‐shelled groups). This challenged traditional hypotheses that proposed that molluscs gradually evolved complex phenotypes from simple, worm‐like animals, a view that is corroborated by developmental studies that showed that aplacophorans are secondarily simplified. Gene expression data indicate that key regulators involved in anterior–posterior patterning (the homeobox‐containing Hox genes) lost this function and were co‐opted into the evolution of taxon‐specific novelties in conchiferans. While the bone morphogenetic protein (BMP)/decapentaplegic (Dpp) signalling pathway, that mediates dorso‐ventral axis formation, and molecular components that establish chirality appear to be more conserved between molluscs and other metazoans, variations from the common scheme occur within molluscan sublineages. The deviation of various molluscs from developmental pathways that otherwise appear widely conserved among metazoans provides novel hypotheses on molluscan evolution that can be tested with genome editing tools such as the CRISPR/Cas9 (clustered regularly interspaced short palindromic repeats/clustered regularly interspaced short palindromic repeats‐associated protein9) system.

## I. INTRODUCTION: THE RISE OF MOLLUSCA

**Figure 1:**
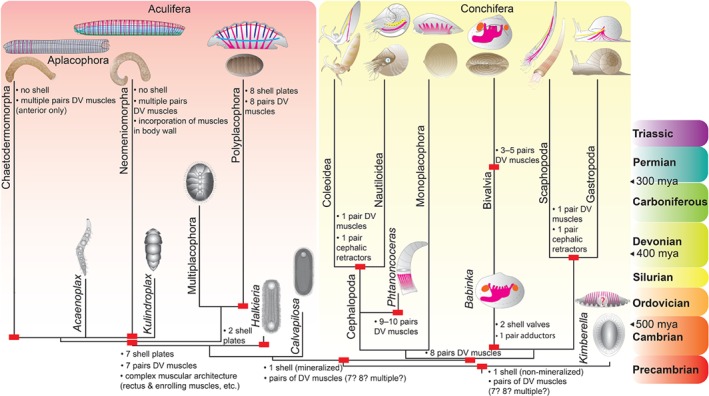
Molluscan intraphyletic relationships and hypothesized evolutionary pathways of major exoskeletal and muscular subsets. Only changes in character states are indicated (red rectangles). Phylogeny based on Smith *et al*. (2011) and Vinther *et al*. (2017). Myoanatomical condition is indicated where known. ‘?’ in *Kimberella* indicates that it is still debated whether or not the respective serial indentations represent fossilized muscle strands. Assuming that *Kimberella* is a stem‐group mollusc, the Aculifera–Conchifera concept favours a single‐shelled ancestor to all molluscs, most likely with serially repeated dorso‐ventral (DV) musculature (magenta). After the split from stem‐group aculiferans, a body plan with seven shell plates and corresponding DV muscles as well as multiple subsets of additional muscles evolved on the line leading to Aculifera. Vermification along with incorporation of distinct muscular units [e.g. ventro‐lateral muscle (green), enrolling muscle (light blue), rectus muscle (red), ring muscles (dark blue)] into the adult body wall occurred in the aplacophorans (potentially several times independently), whereby some extinct taxa maintained the shell armour. Within Polyplacophora, the sevenfold seriality was retained in the extinct multiplacophorans; recent polyplacophorans have secondarily acquired an eighth plate with an additional set of DV muscles. The conchiferans retained the single‐shelled condition and muscular seriality from the molluscan ancestor and probably had eight sets of DV muscles as exhibited by recent monoplacophorans and stem‐group bivalves [that evolved a second shell and two adductor muscles (orange)]. Scaphopods, gastropods, and cephalopods have reduced their DV musculature to one pair and have evolved distinct cephalic retractors (yellow). Following the phylogenetic scenario depicted here, this occurred twice independently. Recent bivalves have retained muscular seriality to a certain degree, with most representatives having 3–5 DV muscles.

Having conquered almost all terrestrial and aquatic habitats on Earth, molluscs are among the best‐known animals on the planet. The exploitation of some representatives as sources of food (snails, mussels, clams, squids, octopuses) or jewellery (e.g. pearls) and, more recently, for biomedical applications [e.g. toxins from cone snails in the treatment of neural diseases such as epilepsy, Parkinson's, Alzheimer's or pain management (Anderson & Bokor, 2012; Romero *et al.,* 2017)] has resulted in considerable commercial value for some species. The almost unmatched diversity of molluscan morphological phenotypes is exemplified by well‐known representatives such as the gastropods (snails, slugs), bivalves (clams, mussels), and cephalopods (nautiluses, squids, octopuses), but also includes more enigmatic groups such as spicule‐bearing, simple worms (the aplacophorans), flattened, ovoid, shell plate‐bearing polyplacophorans (chitons), circular monoplacophorans with a single, cap‐like shell, and the scaphopods (tusk shells), that owe their name to their bent, elephant tooth‐like shell in which the animal resides (Haszprunar & Wanninger, 2012). These dramatic variations in overall body plan morphology render molluscs an ideal group for comparative studies into how evolution has brought about phenotypic diversity from a common ancestor that roamed the oceans' seafloors at least 550 million years ago (mya) (Parkhaev, 2008, 2017; Haszprunar & Wanninger, 2012; Vinther *et al.,* 2012 *a*,*b*; Vinther, 2014, 2015; Wanninger & Wollesen, 2015) (Fig. 1).

The combination of such an ancient evolutionary history together with the occurrence of mineralized exoskeletal hard parts in their body plan has resulted in a rich fossil record, at least of the shell‐bearing taxa (Parkhaev, 2008, 2017). These findings together with molecular clock estimates revealed a picture according to which all major molluscan sublineages are deeply rooted in the Cambrian (Vinther, 2014, 2015) (Fig. 1). Thereby, Aculifera, that comprises the aplacophoran clades Solenogastres (Neomeniomorpha) and Caudofoveata (Chaetodermomorpha) as well as their sister clade Polyplacophora, originated at least 540 mya, i.e. at the Ediacaran–Cambrian border. Its sister taxon, Conchifera, that includes all other molluscs that derived from a uni‐shelled ancestor, emerged approximately 15 my later (Vinther, 2014, 2015) (Fig. 1). If correct, this evolutionary time frame implies that the last common ancestor of all molluscs (LCAM) already lived in the Ediacaran, i.e. before the infamous Cambrian Explosion. However, no Precambrian fossilized exoskeletal elements are known that can be unambiguously assigned to an early mollusc, leaving much room for speculation as to when shell(s) and spicules first arose within the phylum and whether or not the LCAM bore any armour at all.

The enigmatic Ediacaran fossil *Kimberella quadrata* Glaessner & Wade, 1966, originally described in the late 1950s (Glaessner & Daily, 1959) and proposed to represent a Precambrian cnidarian in early studies (Glaessner & Wade, 1966; Wade, 1972), received considerable attention in the decades following its description, because re‐analyses considered it the earliest known bilaterian – and likely a stem‐group mollusc (Fedonkin & Waggoner, 1997; Fedonkin, Simonetta & Ivantsov, 2007; Ivantsov, 2010; Gehling, Runnegar & Droser, 2014; but see, e.g. Parkhaev, 2017 for an alternative view). The fossil specimens found were interpreted as bearing imprints of an elongated, slender foot surrounded by a mantle that is separated from the former by a circumpedal mantle cavity (Seilacher, 1999; Vinther, 2015), thus resembling the gross morphological ventral view of modern polyplacophoran molluscs. Additional proposed shared features between *Kimberella* and recent molluscs mainly concerned characteristics related to the digestive system (Vinther, 2015), as well as extra‐corporal, brush‐like imprints that are commonly found in association with *Kimberella* specimens (Fedonkin, Simonetta, & Ivantsov, 2007; Ivantsov, 2009, 2013; Gehling, Runnegar, & Droser, 2014; Vinther, 2015). These are thought to be trace fossils of a scraping‐type feeding apparatus similar to a molluscan radula (Vinther, 2015; disputed by Parkhaev, 2017). However, despite being heavily chitinized and equipped with teeth that in some cases are impregnated with mineral ions, no traces of a fossilized radula have hitherto been found in any of the dozens of *Kimberella* specimens reported to date (Fedonkin & Waggoner, 1997). No evidence for a mineralized exoskeleton was found, but an apparently rigid epidermal covering was interpreted as an oval (non‐mineralized) shell, and point‐like structures were suggested to constitute putative remains of sclerites (Fedonkin & Waggoner, 1997). If indeed this collection of characters was interpreted correctly, there would remain little doubt concerning the molluscan nature of *Kimberella* and the definite origin of Mollusca in the Precambrian.

Placing *Kimberella* along the molluscan stem line increases the likelihood of a single‐shelled monoplacophoran‐ (i.e. conchiferan‐) and not a polyplacophoran‐like LCAM, most likely with serially arranged dorso‐ventral musculature and an unsegmented body, two features that are also commonly assigned to *Kimberella* (Fedonkin & Waggoner, 1997). Such an ancestral mollusc would also bear striking similarities to a specific larval type of entoprocts (kamptozoans) with serially arranged dorso‐ventral musculature, a distinct, ciliated creeping foot, and a cuticle‐covered dorsal epidermis, lending further support for the morphology‐based Tetraneuralia (formerly Sinusoida or Lacunifera) concept that proposes a sister group relationship of Mollusca and Entoprocta (Bartolomaeus, 1993; Ax, 1999; Wanninger, Fuchs, & Haszprunar, 2007; Haszprunar & Wanninger, 2008; Wanninger, 2009).

The recent description of the flattened, sclerite‐bearing, single‐shelled, proposed aculiferan fossil *Calvapilosa* from the Ordovician shows shared traits with the enigmatic sachitids that include the two‐shelled *Halkieria* and led the authors to suggest that the last common ancestor of Aculifera was single‐shelled (Vinther *et al.,* 2017). This single shell was most likely inherited from the LCAM, a hypothesis well in line with the *Kimberella* concept. While the ur‐aculiferan may well have been single‐shelled [with secondary acquisition of a second plate in halkierids (Vinther *et al.,* 2017)], a holistic consideration of palaeontological, morphological, and developmental data (see below) strongly argues in favour of a last common polyplacophoran–aplacophoran ancestor with seven shell plates and corresponding dorso‐ventral muscle sets that evolved after the split from the proposed stem group aculiferans (*Halkieria*, *Calvapilosa*, and others) (Fig. 1). This sevenfold seriality was retained by some extinct taxa (the multiplacophorans and the aplacophorans *Acaenoplax* and *Kulindroplax*) and is still expressed during ontogeny of recent polyplacophorans and aplacophorans, where seven dorso‐ventral muscle units form first (Wanninger & Haszprunar, 2002; Scherholz *et al.,* 2013, 2015). From this common ancestor, the eight‐fold adult seriality evolved secondarily in recent polyplacophorans, while recent aplacophorans underwent body simplification and vermification accompanied by loss of the shell armour (Fig. 1).

Irrespective of whether or not *Kimberella* is an early stem‐line mollusc and whether or not other debated fossils such as *Acaenoplax*, *Wiwaxia*, *Halkieria*, or the newly described *Calvapilosa* belong to the molluscan stem, are members of later‐branching molluscan sublineages, or do not constitute molluscs at all (Conway Morris, 1985; Butterfield, 1990; Conway Morris & Peel, 1990, 1995; Steiner & Salvini‐Plawen, 2001; Sutton *et al.,* 2001, 2004; Scheltema & Ivanov, 2002; Vinther & Nielsen, 2005; Conway Morris & Caron, 2007; Telford & Budd, 2011; Sutton & Sigwart, 2012; Vinther, 2014; Parkhaev, 2017; Vinther *et al.,* 2017), a sound inference of molluscan evolutionary history involving ground pattern reconstruction requires a well‐supported phylogenetic framework (Wanninger, 2015). While this has traditionally been a major gap in molluscan research, both concerning intraspecific relationships of class‐level taxa but also with respect to unresolved molluscan sister group relationships (Wanninger, 2009; Haszprunar & Wanninger, 2012), recent large‐scale phylogenomic analyses (Kocot *et al.,* 2011; Smith *et al.,* 2011) lend hope that some emerging phylogenetic patterns within the phylum will consolidate and will provide a long‐sought base for addressing questions concerned with molluscan origins and phenotypic diversification.

The recent progress in molluscan phylogenetics is supplemented by numerous comparative developmental studies at the morphological, cellular, and molecular levels that have provided significant insights into lineage‐specific evolutionary pathways concerning body plan formation, the emergence of morphological novelties, and conserved *versus* newly acquired (co‐opted) gene functions at various taxonomic levels. In the following, the major recent advances in molluscan phylogenetics and developmental biology are outlined and interpreted in an integrated evolutionary framework. In addition, remaining key questions and hypotheses are pointed out that can now be addressed with the aid of state‐of‐the‐art technologies.

## II. A NOVEL VIEW ON MOLLUSCAN PHYLOGENY

**Figure 2:**
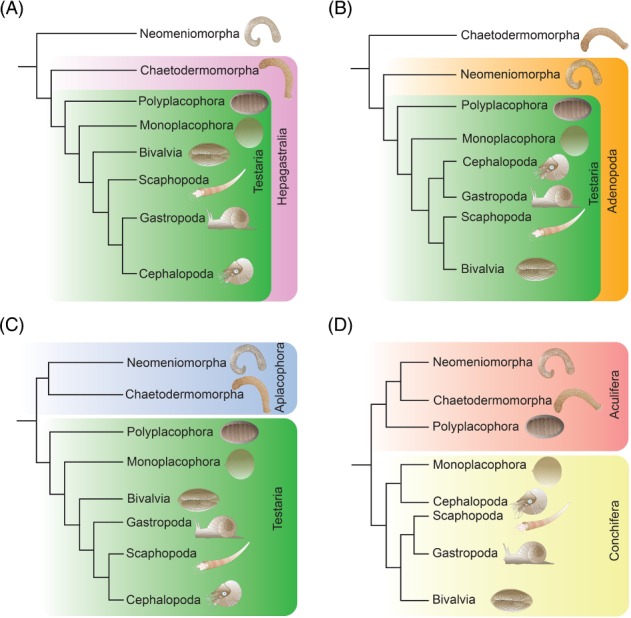
Major competing phylogenetic scenarios for Mollusca. Note that Conchifera is monophyletic in all cases but its internal relationships are highly debated. (A) Hepagastralia–Testaria concept with Neomeniomorpha as the earliest offshoot; (B) Adenopoda–Testaria concept with Chaetodermomorpha as the earliest offshoot; (C) Aplacophora–Testaria concept with a monophyletic assemblage of Polyplacophora and Conchifera (Testaria) as sister to Aplacophora; and (D) Aculifera–Conchifera concept, according to which Polyplacophora together with Aplacophora form the Aculifera that is the sister clade to Conchifera. Note that scenarios A and B suggest a gradual increase in body plan complexity from the vermiform neomeniomorphs and chaetodermomorphs, a situation that is not supported by paleontological and developmental data. By contrast, the basal dichotomy in C and D and the unresolved molluscan sister group relationship does not allow for an unambiguous reconstruction of molluscan ancestry without the addition of fossil and developmental data.

Molluscan interrelationships have been contentious for a long time. In the pre‐genomic era, cladistic analyses based on morphological data sets largely agreed on a phylogenetic scenario that placed the aplacophoran taxa (Neomeniomorpha and Chaetodermomorpha) as the earliest extant offshoots, either with a monophyletic Aplacophora as sister to the remaining molluscs (Testaria), of which Polyplacophora was considered the sister clade to all primarily single‐shelled forms (Conchifera) (Waller, 1998), or with either of the two aplacophoran groups as sister to the remaining class‐level taxa (Salvini‐Plawen, 1972, 1991; Haszprunar, 1992, 2000; Salvini‐Plawen & Steiner, 1996, 2014) (Fig. 2). This latter situation thus also recovered a monophyletic Testaria and Conchifera, and in the majority of cases proposed Neomeniomorpha as the earliest molluscan branch, rendering Chaetodermomorpha and Testaria a monophylum termed Hepagastralia (Haszprunar, 2000) (Fig. 2). Most proponents of any of these scenarios, in particular those favouring the Hepagastralia concept, suggested a gradual increase in body‐plan complexity from a simple vermiform, aplacophoran‐like ancestor towards the more complex conchiferans, usually terminating with the cephalopods as the most ‘advanced’ representatives.

At that time, morphological data had largely been restricted to adult life‐cycle stages for most of the crucial class‐level taxa. Solid ontogenetic (morphogenetic) data for both aplacophoran taxa, the polyplacophorans, the scaphopods, as well as the basally branching bivalve and gastropod lineages were virtually non‐existent (Wanninger & Wollesen, 2015). This lack of knowledge on potential transitory characters that are restricted to given ontogenetic stages led to incomplete data matrices for the morphology‐based phylogenetic analyses and also hampered sound ground‐pattern reconstruction (Wanninger, 2015).

The advent of molecular data as a source to resolve organismal interrelationships and the rapid increase in speed and quality of sequence data and enhanced *in silico* applications, together with joint efforts by large international consortia, have resulted in a plethora of suggested cladograms aiming to reconstruct animal phylogeny at various taxonomic levels (Dunn *et al.,* 2008; Hejnol *et al.,* 2009; Struck *et al.,* 2011; Misof *et al.,* 2014). For the phylum Mollusca, however, highly conflicting and unconvincing intraphyletic topologies prevailed (Haszprunar & Wanninger, 2012), despite the use of increasingly large molecular data sets such as, e.g., ribosomal protein‐encoding genes (e.g. Meyer, Witek, & Lieb, 2011). One surprising hypothesis that emerged was based on a collection of 16S rRNA, 18S rRNA, 28S rRNA, histone H3, and cytochrome oxidase I (COI) sequences and found that the sole monoplacophoran species included in the study clustered within Polyplacophora, leading the authors to suggest a monophyletic Serialia (Polyplacophora + Monoplacophora) (Giribet *et al.,* 2006). The most parsimonious explanation of such a scenario implies that the serial arrangement of organ systems such as the gills and the eight sets of complex dorso‐ventral muscles, shared by polyplacophorans and monoplacophorans, evolved along the line leading to the serialians and not at the molluscan base. Admittedly, in addition to poor taxon sampling for crucial clades such the aplacophorans and the monoplacophorans and an unrecognized chimeric monoplacophoran 28S rRNA sequence in the data set, there were several additional problems with the cladogram presented. These included poor jackknife support for the monophyly of Mollusca, diphyly of Gastropoda and Bivalvia, and a sister group relationship of Cephalopoda and Chaetodermomorpha. Although some subsequent analyses that were corrected for the chimeric 28S rRNA sequence and enjoyed increased taxon sampling also recovered Serialia (Wilson, Rouse, & Giribet, 2010; Kano *et al.,* 2012; Stöger *et al.,* 2013), this hypothesis is currently not widely favoured, probably also because the remaining nodes within Mollusca remained largely unresolved.

The vivid discussions on molluscan phylogeny and ancestry were additionally fuelled by two influential studies published simultaneously in 2011 (Kocot *et al.,* 2011; Smith *et al.,* 2011) (Fig. 2D). Both analyses used large‐scale phylogenomic data sets and, independent of each other, proposed a scenario with a basal dichotomous split, separating Mollusca into two early lineages, Aculifera (Neomeniomorpha + Chaetodermomorpha as monophyletic Aplacophora being sister to Polyplacophora) and Conchifera. Despite considerable attention, this scenario was not entirely new but rather a confirmation of an earlier morphology‐based hypothesis, in which the aplacophorans had already been regarded as secondarily derived by progenesis from a polyplacophoran‐like ancestor (Scheltema, 1993, 1996).

Of the two phylogenomic studies mentioned above, only one included all eight class‐level molluscan taxa (Smith *et al.,* 2011), with Monoplacophora missing in the other (Kocot *et al.,* 2011). The more complete analysis (Smith *et al.,* 2011) found a surprising monophylum (Monoplacophora + Cephalopoda) as sister to ((Gastropoda + Scaphopoda) + Bivalvia), while the other one (Kocot *et al.,* 2011) proposed Cephalopoda as sister taxon to the remaining conchiferans, a scenario somewhat compatible with the monoplacophoran–cephalopod situation, given the lack of the monoplacophorans in this work. Herein, Scaphopoda was recovered as sister to (Gastropoda + Bivalvia). The incongruencies concerning the inner‐conchiferan topologies still remain and render assessments regarding the nature of the last common conchiferan ancestor difficult, thus also posing problems for ground pattern reconstructions of the LCAM. The joint recovery of a basal split of Mollusca into Conchifera and Aculifera with a monophyletic Aplacophora, however, is a significant step forward and provides a long‐sought framework for reconstructing deep evolutionary scenarios for the phylum and some of its key lineages. Given that these two analyses are based on the largest molecular data sets by far, and owing to their agreement in at least some crucial intraphyletic relationships, the Aculifera–Conchifera concept currently enjoys wide acceptance.

## III. EVODEVO, GENOMES, AND THE DIVERSIFICATION OF MOLLUSCS

### (1) Molluscan organogenesis and body plan evolution

#### (a) *Shells*

Shells are the most conspicuous features of molluscs. Yet, their evolutionary origin has been a matter of debate for many decades (Haas, 1981; Kniprath, 1981; Wanninger & Wollesen, 2015). Molluscan shells are usually made of calcium carbonate and are covered by an organic matrix, the periostracum. While there remains little doubt about a common evolutionary origin of the conchiferan adult shell (teleoconch) that was inherited from a uni‐shelled last common ancestor, several studies have revealed that the molecular underpinnings, in particular of bivalve and gastropod shells, are relatively complex (Jackson, Wörheide, & Degnan, 2007; Nudelmann, 2015) and show striking differences among taxa, even among species (Aguilera, McDougall, & Degnan, 2017). As such, it could be shown that a nacreous (mother of pearl) layer evolved multiple times both between but also within gastropods and bivalves (Jackson *et al.,* 2010). An often‐ignored phenomenon in the discussion on conchiferan shell evolution is the fact that there are essentially three types of shells that are difficult to homologize: the embryonic shell (protoconch I or prodissoconch I, often misunderstandingly termed ‘larval shell’), the actual larval shell (proto‐ or prodissoconch II), and the adult shell (teleoconch) (Bandel, 1975). The protoconch I constitutes the first‐formed shell that is typical to conchiferans that undergo indirect development *via* trochophore‐ and veliger‐type larvae (i.e. all scaphopods and bivalves, many marine gastropods). This is the type of shell that derives from a dorsal shell field that forms by invagination and subsequent evagination of ectodermal cells (Kniprath, 1981; Wanninger & Wollesen, 2015). Secretion of the embryonic shell is rapid and results in a homogeneous, smooth, colourless protoconch I. Both other shell types, the protoconch II (larval shell; only present in some bivalves and caenogastropods) and, importantly, the adult shell (teleoconch) form continuously by secretory cells from the mantle margin as long as the animal grows. Both types may be richly sculptured and the teleoconch may exhibit intricate colour patterns and an additional inner nacreous layer. Accordingly, it is important to discriminate between these shell types in any discussion on conchiferan shell development and evolution, particularly if gene expression signatures among taxa are compared. Matters get even more complicated if the polyplacophoran shell plates are included in the discussion, because here the cellular dynamics and the secretion process as well as the cell lineage differ considerably from the conchiferan‐type shells (Kniprath, 1980; Haas, 1981; Henry, Okusu, & Martindale, 2004).

Several common developmental regulators have been shown to be involved in the formation of the embryonic shell field. One important player is the transcription factor *Engrailed* that demarcates the conchiferan shell field from other mantle tissue (Jacobs *et al.,* 2000; Wanninger & Haszprunar, 2001; Nederbragt, Van Loon & Dictus, 2002). *Dpp*, *Grainyhead*, *Ferritin*, and *CS1* have been shown to play a role in shell field formation in bivalves and *Hox1*, *Post1*, *Post2*, and *Calmbp I* are expressed in the embryonic shell field of gastropods (the latter three also in the mantle margin that secretes the adult shell) (Jackson *et al.,* 2007). The most complete survey of genes involved in molluscan shell formation is based on an analysis of the oyster genome (Zhang *et al.,* 2012), where 259 shell proteins were found. These included chitin as well as a fibronectin‐like protein and chitin synthase that are both highly expressed during embryonic shell field formation. Fibronectin together with laminin and several collagens were found at high concentrations in adult mantle tissue. Since these proteins constitute important components of the extracellular matrix across metazoans, it was proposed that the organic matrix of the oyster shell bears similarities to connective tissue of other animals (Zhang *et al.,* 2012). The diverse protein composition of the adult oyster shell is further enhanced by a large set of proteinases and proteinase inhibitors that may interactively support formation and modification of the organic matrix of the shell. Dozens of additional genes and proteins have been found in the secretome of the mantle of various gastropods and bivalves, many of them species‐specific (Jackson *et al.,* 2010; Fang *et al.,* 2011; Marie *et al.,* 2012; Aguilera *et al.,* 2017), but a broader comparison throughout the conchiferans in an evolutionary framework is yet difficult due to the lack of detailed data for other clades.

#### (b) *Musculature and seriality*

Together with the recent advances in molluscan phylogeny including the revived Aculifera–Conchifera concept, a wide array of novel data on molluscan genomics and comparative development (EvoDevo) have become available, providing an important window into evolutionary pathways and common ground patterns of various lineages. Accordingly, it was shown that during ontogeny, neomeniomorph aplacophorans recruit their body wall muscles from a complex arrangement of larval muscular subsets that are gradually incorporated into the adult tube‐like body (Scherholz *et al.,* 2013, 2015). A number of these larval muscle systems are shared exclusively by neomeniomorphs and polyplacophorans (the chaetodermomorph condition is still unknown) and include a laterally positioned enrolling muscle, a dorsal rectus muscle that spans the anterior–posterior axis of the animal, as well as several ventral longitudinal systems (Fig. 1). While most of these muscular units are retained and elaborated in adult polyplacophorans, they are largely remodelled and incorporated into the developing postmetamorphic body wall musculature of neomeniomorphs (Scherholz *et al.,* 2013, 2015). Interestingly, both, juvenile polyplacophorans and neomeniomorph larvae show a transitory stage of a sevenfold seriality in their dorso‐ventral musculature (Wanninger & Haszprunar, 2002; Scherholz *et al.,* 2013, 2015). While in the former the eighth set is added together with the remaining posterior shell plate a considerable time after metamorphosis, multiple pairs are added to the seven primary dorso‐ventral muscles in postmetamorphic neomeniomorphs.

A transitory sevenfold seriality shared by aplacophorans and polyplacophorans is also known from chaetodermomorph larvae that show seven rows of epidermal papillae (Nielsen *et al.,* 2007), as well as from a reported neomeniomorph ‘postlarva’ with seven rows of spicules (Scheltema & Ivanov, 2002). This is congruent with the fossil record, from which aplacophorans (!) with seven shell plates (Sutton *et al.,* 2004, 2012) as well as a sub‐group of polyplacophorans, the multiplacophorans, with 17 shell plates arranged in seven (!) rows (Vendrasco, Wood, & Runnegar, 2004; Vinther *et al.,* 2012 *a*), have been described. Taken together, these data show that the last common aculiferan ancestor had an overall polyplacophoran‐like morphology with a suite of highly complex muscle systems and a series of seven dorso‐ventral muscles, most likely accompanied by seven shell plates (Fig. 1). The multiplacophorans and some (extinct) aplacophorans (*Kulindroplax* and *Acaenoplax*) retained this sevenfold arrangement of shell plates (multiplied in rows 2–6 in multiplacophorans), while extant polyplacophorans acquired an eighth plate secondarily and recent aplacophorans lost their shell plates altogether (Fig. 1). The cylindrical anatomy of aplacophorans constitutes a secondary condition that evolved along with the integration of the individual longitudinal muscle sets into the body wall that eventually resulted in their worm‐like phenotype (Scherholz *et al.,* 2013, 2015). Notably, secondary vermification is not limited to the aculiferans but is a recurring phenomenon within molluscan sublineages, e.g., in heterobranch gastropods (e.g. *Rhodope*, *Helminthope*; Brenzinger, Wilson, & Schrödl, 2011; Brenzinger, Haszprunar, & Schrödl, 2013) and teredinid bivalves (shipworms) (Turner, 1966).

While the picture of aculiferan evolution and diversification appears to become clearer owing to integrative data sets from various disciplines, the conchiferan condition remains blurry. Not only are conchiferan interrelationships still highly controversial (Haszprunar & Wanninger, 2012; Schrödl & Stöger, 2014), but the vast phenotypic diversity of its individual class‐level clades renders ground‐pattern reconstruction difficult no matter what kind of topology will eventually be agreed on. With their simple single shell and repetitive organ systems including gills, nephridia, commissures, and, most importantly, eight sets of dorso‐ventral muscles, the monoplacophorans intuitively make good candidates to be directly compared to the aculiferan condition. Interestingly, fossil bivalves with eight (McAlester, 1965) and nautiloid cephalopods with 9 or 10 (Kröger & Mutvei, 2005) sets of dorso‐ventral muscles are known, while most recent conchiferans only have one (bivalves often have three) (Haszprunar & Wanninger, 2000) (Fig. 1). In the light of these findings one may be tempted to propose a gradual ‘de‐serialization’ within the Conchifera, starting with a *Kimberella*‐monoplacophoran‐like ancestor and terminating with one single dorso‐ventral muscle in derived gastropods and recent cephalopods (Haszprunar & Wanninger, 2000). However, the uncertainties in conchiferan phylogeny currently hamper a final conclusion. Moreover, it must be borne in mind that muscular development and evolution appears to be a dynamic process in molluscs with a considerable degree of plasticity, rendering assessments of homologous traits *versus* homoplasies problematic. In addition, details on monoplacophoran ontogeny are still entirely lacking and it thus remains unknown as to how adult seriality develops in this clade and whether or not informative transitory elements occur during development (Wanninger & Wollesen, 2015).

### (2) Molluscan body axes and morphological novelties

Alongside the emerging patterns of molluscan phenotypic evolution based on morphogenetic and palaeontological data, significant insights have been gained concerning the molecular mechanisms that govern their development (Wanninger & Wollesen, 2015). Thereby, the release of the genomes of various gastropods including the Pacific abalone *Haliotis discus hannai* (Nam *et al.,* 2017), the freshwater snail *Biomphalaria glabrata* (Adema *et al.,* 2017), and the basally branching owl limpet *Lottia gigantea* (Simakov *et al.,* 2013) together with several bivalves [e.g. *Crassostrea gigas* (Thunberg, 1793) (Zhang *et al.,* 2012), *Patinopecten yessoensis* (Jay, 1857) (Wang *et al.,* 2017), and other scallop, oyster, and mussel species (Takeuchi *et al.,* 2012; Du *et al.,* 2017; Li *et al.,* 2017, 2018; Sun *et al.,* 2017)] and the California two‐spot octopus *Octopus bimaculoides* Pickford & McConnaughey, 1949 (Albertin *et al.,* 2015) provided an important framework for studies into key developmental regulators such as Hox and ParaHox genes as well as signalling molecules involved in dorso‐ventral axis patterning (bone morphogenetic protein (BMP)/decapentaplegic (Dpp) pathway) and left/right determination (Nodal pathway).

#### (a) *Hox genes, anterior–posterior axes, and molluscan innovations*

**Figure 3:**
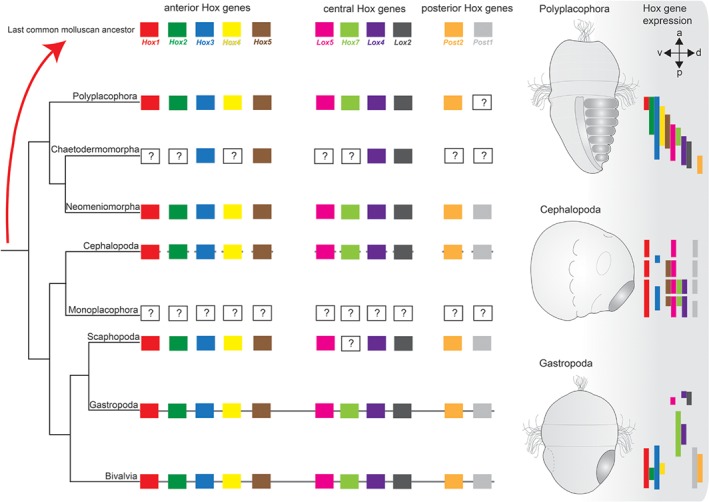
Molluscan Hox genes and their expression patterns in polyplacophorans, cephalopods, and gastropods. Except for Gastropoda, Bivalvia, and Cephalopoda, for which genomes are available, Hox sequences have only been identified from transcriptomic data sets. Thus, uncertainties exist concerning the definite lack of individual Hox genes in chaetodermomorphs, polyplacophorans, and scaphopods, indicated by ‘?’. No monoplacophoran Hox genes are known to date. Note that for most taxa data were combined from different species. A continuous line between Hox gene symbols indicates an intact Hox cluster for the respective taxon (note that for bivalves, both intact and disrupted Hox gene arrangements are known). The discontinuous line in Cephalopoda indicates a disrupted Hox cluster. The absence of a line in Polyplacophora, Chaetodermomorpha, Neomeniomorpha, and Scaphopoda indicates that the Hox gene arrangement is unknown. Colour‐coded bars show gene expression along the anterior–posterior axis of a mid‐stage polyplacophoran trochophore larva, an early cephalopod embryo, and a late‐stage gastropod trochophore larva. For clarity, expression in specific morphological features is not shown. Note that a staggered expression of Hox genes, as predicted by the concept of colinearity, is found only in polyplacophorans. Shell field(s) in the polyplacophoran and gastropod larvae as well as the mantle *anlage* in the cephalopod embryo are shaded in dark grey. Abbreviations: a, anterior; d, dorsal; p, posterior; v, ventral.

Hox gene expression surveys across several molluscan representatives showed that in polyplacophorans, these genes are expressed in a staggered mode along the anterior–posterior body axis, as predicted by the proposed ancestral colinear mode of Hox gene expression in bilaterians (Fritsch *et al.,* 2015; Fritsch, Wollesen & Wanninger, 2016) (Fig. 3), although their arrangement in the polyplacophoran genome is still unknown. Surprisingly, such a staggered mode of expression was not found in the sole gastropod *Gibbula varia* (Linnaeus, 1758) for which the full set of the 11 molluscan Hox genes was investigated (Samadi & Steiner, 2009, 2010) (Fig. 3), despite the fact that in the patellogastropod *Lottia gigantea* the Hox genes form a homogeneous cluster (Simakov *et al.,* 2013) (Hox arrangement in *Gibbula* remains elusive). Instead of being confined to domains along the anterior–posterior axis, *Gibbula* Hox genes are expressed highly structure‐specific and were found in cells of the larval apical organ, the foot, the shell field, and the prototroch (Samadi & Steiner, 2009, 2010). This provided evidence that, at least in this gastropod, Hox genes have lost their ancestral role in anterior–posterior axis patterning and have acquired novel functions to form trochozoan‐ and mollusc‐specific characters, supporting the general view that variations in gene expression patterns and gene interactions rather than the evolution of novel genes are the prime driving forces in the evolution of morphological diversity (Davidson, 2006; Carroll, 2008). However, aside from the recruitment of existing genes into novel functions, lineage‐specific genes and gene family expansions that account for various ecological and functional needs (e.g. protocadherins, zinc‐finger transcription factors of the cysteine‐2/histidine‐2 (C2H2) superfamily, components of various metabolic pathways, as well as heat shock and shell matrix proteins) are also known for molluscs, e.g. from the octopus, the limpet, as well as from the Pacific and the pearl oyster (Simakov *et al.,* 2013; Albertin *et al.,* 2015; Paps *et al.,* 2015; Takeuchi *et al.,* 2016).

The finding of non‐colinear Hox gene expression in gastropods corroborated an earlier study on the Hawaiian bobtail squid, *Euprymna scolopes*, where Hox genes similarly were not found to contribute to axial patterning but rather were expressed in distinct organ systems including the gills, arms, funnel, and light organ (Lee *et al.,* 2003) (Fig. 3). Again, the genomic architecture of this species still awaits publication, but the recently released genome of the octopus *Octopus bimaculoides* showed an entirely disrupted Hox cluster, where the various sequences are placed in distant regions on the genome (Albertin *et al.,* 2015). Screening of the *Octopus* genome also revealed hundreds of novel genes that are expressed at high levels in cephalopod‐specific structures such as the suckers, skin, or certain neural components. This, together with the finding that in *Octopus* considerable genome shuffling and expansion of individual gene families such as zinc‐finger proteins, chitinases, G‐protein‐coupled receptors, or protocadherins has taken place [whereby the overall content of gene families is not significantly larger compared to other invertebrates (Albertin *et al.,* 2015)], indicates that the emergence of the complex cephalopod body plan was likely due to a combination of mechanisms including the evolution of novel genes, multiplication of individual genes, as well as loss‐of‐function and acquisition of novel functions of conserved gene networks. Importantly, a recent study found that unlike other bilaterians, coleoids, i.e. all cephalopods except for nautiluses, diversify their proteomes to a hitherto unknown extent by RNA editing (Liscovitch‐Brauer *et al.,* 2017). This recoding appears to be evolutionarily conserved and adaptive among coleoids and may be one reason for their sophisticated cognitive abilities.

As with *Octopus*, analyses of the genome of four bivalves, namely the Pacific oyster *Crassostrea gigas*, the pearl oyster *Pinctada fucata* (Gould, 1850), and two deep‐sea mussels, showed a disorganized arrangement of the Hox genes (Zhang *et al.,* 2012; Sun *et al.,* 2017; Wang *et al.,* 2017). In *C. gigas* and *P. fucata*, the Hox cluster appeared to be split into four or five distinct regions which are all framed by non‐Hox sequences (Zhang *et al.,* 2012; Wang *et al.,* 2017). Quantitative expression analyses of *Crassostrea gigas* stages are in line with the split Hox cluster insofar as no temporal correlation was found between expression of a given Hox gene in a certain ontogenetic stage and its relative position to other Hox genes on the genome (Zhang *et al.,* 2012). However, in two other bivalves, the scallops *Patinopecten yessoensis* and *Chlamys farreri*, the Hox genes do appear to form a true cluster (Li *et al.,* 2017; Wang *et al.,* 2017). While precise tempo‐spatial expression analyses of Hox genes spanning entire embryonic and larval development are still lacking, transcript localization in gastrulae of *Patinopecten yessoensis* suggests some degree of staggered expression of four Hox genes (*Hox1*, *Hox4*, *Lox5*, *Post2*) in this stage, and quantitative analyses revealed staggered temporal expression of individual Hox genes within four (virtual) *Patinopecten yessoensis* subclusters (Wang *et al.,* 2017). This calls for further detailed positional mapping of Hox transcripts in crucial developmental stages by *in situ* hybridization analyses to assess the degree to which bivalves have retained the polyplacophoran‐like anterior–posterior axial expression gradient and/or whether they (also) follow the gastropod–cephalopod pathway of organ‐specific Hox gene expression.

Taken together, molluscs, and in particular the conchiferans, appear to show a complex interplay employing multiple changes in genomic architecture and gene functions that most likely contributed to the evolution of lineage‐specific morphological novelties. The expected release of additional genomes in the near future will provide an important resource to test which of these molecular mechanisms have contributed to the various apomorphic features of individual molluscan subtaxa.

#### (b) *The Dpp/BMP pathway and dorso‐ventral patterning*

Bilaterian animals are not only characterized by a distinct anterior–posterior axis but also by defined dorsal and ventral body regions. The underlying regulatory network that commonly determines dorso‐ventral polarity is the Dpp/BMP signalling pathway (De Robertis & Sasai, 1996; Ferguson, 1996; De Robertis & Kuroda, 2004; Ashe & Briscoe, 2006; Lowe *et al.,* 2006; Cebria, Salo, & Adell, 2015). Briefly, the morphogen‐encoding gene *Dpp* (the invertebrate homolog of the vertebrate *Bmp2/4*) is largely expressed dorsally in protostome animals, while its antagonists such as *Chordin* or *Noggin* have a predominantly ventral expression domain (Tan, Huan & Liu, 2017). This system is inverted in chordates with ventral *BMP* expression and dorsal expression of the antagonists. In extracellular regions of higher Dpp/BMP concentration, these proteins bind to receptors on the cell surface, resulting in the phosphorylation of so‐called SMADs (proteins related to the *Drosophila* mothers against decapentaplegic and the *Caneorhabditis elegans* small worm phenotype protein families), that subsequently migrate into the cell nucleus where they activate downstream target genes (Anderson & Darshan, 2008). One result of this gene/protein cascade is, among others, the formation of the longitudinal neural cords of the central nervous system in regions of suppressed Dpp/BMP signalling, i.e. where the concentration of Dpp/BMP antagonists is high, thus defining the dorsal side of chordates and the ventral side of protostomes (Miller‐Bertoglio *et al.,* 1997; Furthauer, Thisse, & Thisse, 1999; Hild *et al.,* 1999; Kondo, 2007). In molluscs, *Dpp* has been shown to be expressed in the shell field of bivalves (Kin, Kakoi, & Wada, 2009; Tan, Huan, & Liu, 2017) and gastropods (Nederbragt, Van Loon, & Dictus, 2002; Koop *et al.,* 2007; Iijima *et al.,* 2008; Hashimoto, Kurita, & Wada, 2012), thus in dorsal ectodermal domains similar to other protostomes. However, since no further data were available, the question whether these expression patterns hint towards a novel function of *Dpp* in molluscan shell formation or whether *Dpp* is (also) involved in establishing neural and dorso‐ventral identity remained elusive. To this end, a recent experimental study on the model gastropod *Ilyanassa obsoleta* revealed that *Dpp* is intensely expressed on the dorsal side and co‐localized with phosphorylated SMADs, indicating that BMP‐signalling is functional on the dorsal side (Lambert *et al.,* 2016). When *Dpp* was knocked down, no dorsal identity developed, resulting in a rather ‘ventralized’ embryo. Surprisingly, however, and different to other protostomes, ectopic activation of the Dpp/BMP pathway led to the formation of additional neural tissues in the gastropod and not to their repression, as would have been predicted from findings in other bilaterian animals (Lambert *et al.,* 2016). This would suggest that the Dpp/BMP pathway in *Ilyanassa obsoleta* comprises a combination of ancestral (specification of the dorso‐ventral axis) and novel (induction of neuroectoderm formation) functions. Since no further data are available for other molluscan taxa, it is currently impossible to assess the broader implications of these findings for molluscan body plan evolution. However, the study demonstrates that, similar to the Hox genes, the Dpp/BMP pathway seems to exhibit a certain degree of plasticity within Mollusca.

The adult scaphopod and cephalopod body plans have traditionally been thought to have evolved by secondary elongation of the dorso‐ventral axis, probably from a monoplacophoran‐like ancestor, which eventually became dominant over the anterior–posterior one (Naef, 1928; Yochelson, Flower, & Webers, 1973; Kröger, Vinther, & Fuchs, 2011). Expression pattern analyses and experiments involving components of the Dpp/BMP pathway similar to that performed in *Ilyanassa obsoleta* may provide cues in favour or against this classical hypothesis of molluscan phenotypic evolution.

#### (c) *The Nodal pathway and body plan asymmetries*

Although bilaterian animals are by definition characterized by a single primary symmetry plane, many representatives are not symmetrical at all with respect to the morphology and position of certain organ systems in their body. In molluscs, body asymmetry is most obvious in gastropods, where ontogenetic torsion – a process where the body region comprising the head and foot rotates by 180° relative to the mantle cavity – results in a U‐shaped gut, intercrossing visceral nerve cords (streptoneury), and an anteriorly positioned mantle cavity (Wanninger *et al.,* 1999; Wanninger, Ruthensteiner, & Haszprunar, 2000; Page, 2006). This event has been partly reversed during the evolution of heterobranch gastropods, resulting in an asymmetrically positioned mantle cavity, heart, gills, hindgut, and other features, usually on the right side (dextrally, i.e. clockwise‐coiling individuals). Early work on the pulmonates *Biomphalaria glabrata* (ram's horn snail) and *Lymnaea stagnalis* (pond snail), that both have sinistrally and dextrally coiling individuals, revealed that handedness is maternally inherited (Boycott & Diver, 1923; Sturtevant, 1923; Boycott *et al.,* 1930), with the dextral phenotype being dominant over the sinistral one (Shibazaki, Shimizu, & Kuroda, 2004; Liu *et al.,* 2015). As predicted by studies on deuterostomes, asymmetric expression of genes of the Nodal signalling pathway, *Nodal* and *Pitx*, was found in various gastropods depending on their chirality: in the dextral limpet *Lottia gigantea*, both genes are exclusively expressed on the right side, while in sinistral *Biomphalaria glabrata* specimens both genes are expressed on the left side (Grande & Patel, 2009). This finding is consistent with studies on two other gastropods, *Ilyanassa obsoleta* (Say, 1822) and *Crepidula fornicata* (Linnaeus, 1758), where mRNAs are asymmetrically distributed already at early cleavage stages (Lambert & Nagy, 2002; Henry *et al.,* 2010; Rabinowitz & Lambert, 2010). Inhibition of Nodal signalling before the blastula stage in *Biomphalaria glabrata* resulted in loss of *Pitx* expression and non‐chiral individuals with an uncoiled, tubular shell (Grande & Patel, 2009). Experimental manipulation of the genetically determined spiral cleavage program in *Lymnaea stagnalis* (by shifting the micromeres by 90° at third cleavage and thereby artificially producing sinistralized and dextralized embryos, respectively) was accompanied by reversed *Nodal/Pitx* expression. The resulting fertile females maintained the externally imposed chirality but in turn produced offspring with the genetically determined handedness and not the one forced upon their mothers (Kuroda *et al.,* 2009).

A detailed look into the earliest symmetry‐breaking events upstream of Nodal signalling revealed that in *Lymnaea stagnalis* one of two identified diaphanous‐related formin genes, *Ldia2*, is expressed asymmetrically as early as in two‐cell‐stage embryos (Davison *et al.,* 2016). The definite role of formin in establishing chirality in *Lymnaea stagnalis* was confirmed by inhibitory experiments applied to genetically dextral embryos after the second cleavage, whereby the formin‐dependent formation of actin‐containing components of the cytoskeleton was disrupted. This resulted in embryos with four, non‐chirally arranged micromeres similar to wild‐type sinistral embryos (Davison *et al.,* 2016). These findings demonstrate that chirality is already established at a molecular level in this gastropod in early cleaving embryos and thus long before morphologically detectable asymmetries occur. *Dia* was also found to be involved in left–right patterning in the African clawed frog, *Xenopus laevis*, thus suggesting a conserved role of formin in establishing chirality in bilaterians (Davison *et al.,* 2016). Interestingly, however, in two other pulmonate snail genera where both chiral patterns do occur, *Euhadra* and *Partula*, *dia* is not involved in left–right patterning (Davison *et al.,* 2016). This points towards a complex regulatory network underlying the establishment of body plan asymmetries in gastropods. Importantly, these findings once more confirm the commonly emerging picture that developmental pathways and gene functions appear to be highly plastic in molluscs, with a strong tendency towards co‐option as well as loss‐of‐function events occurring even at low hierarchical taxonomic levels.

## IV. PERSPECTIVES

With their origins nested deeply within the Ediacaran, molluscs have a lively and fascinating evolutionary history. Recent advances in palaeontological, phylogenetic, developmental, and experimental approaches have revealed some of the mysteries revolving around the emergence of the large phenotypic diversity encountered in modern and fossil species. The recent establishment of genomic editing tools such as the CRISPR/Cas (clustered regularly interspaced short palindromic repeats/clustered regularly interspaced short palindromic repeats‐associated protein) system in molluscs (Perry & Henry, 2015), together with the ever‐increasing genomic (Zhang *et al.,* 2012; Simakov *et al.,* 2013; Albertin *et al.,* 2015; Li *et al.,* 2017; Wang *et al.,* 2017) and transcriptomic resources (De Oliveira *et al.,* 2016; Li *et al.,* 2017), provide an ideal base to finally pursue questions concerned with molluscan functional genetics. To this end, some molluscan species have already been demonstrated to be particularly amenable to becoming true laboratory models, including the gastropods *Ilyanassa obsoleta* (Goulding & Lambert, 2016) and *Crepidula fornicata* (Henry & Lyons, 2016). Moreover, protocols for comparative studies into morphogenesis and gene expression are available for various representatives distributed all over the molluscan tree, including aplacophorans (Redl *et al.,* 2014, 2016, 2018; Scherholz *et al.,* 2017), polyplacophorans (Fritsch et al., 2015; Wollesen *et al.,* 2015b, 2017; Fritsch, Wollesen, & Wanninger, 2016), scaphopods (Wollesen *et al.,* 2015a), bivalves (Kakoi *et al.,* 2008; Kin *et al.,* 2009; Wollesen *et al.,* 2015b; Pavlicek, Schwaha, & Wanninger, 2018), and cephalopods (Wollesen *et al.,* 2010, 2014, 2015b). Newly obtained sequence data especially for the notoriously undersampled aplacophorans, polyplacophorans, scaphopods, and monoplacophorans should further aid in achieving a better and more stable resolution of molluscan inter‐ and intrarelationships (particularly within Conchifera) as an important prerequisite for evolutionary inferences.

## V. CONCLUSIONS

1. The currently favoured scenario of a deep dichotomy that partitions Mollusca into the monophyletic Aculifera and Conchifera, together with comparative ontogenetic analyses, have shown that the worm‐like aplacophorans derive from a complex ancestor and have only secondarily acquired their simple, vermiform body plan.
2. Comparative studies, in particular of the aculiferans, at various levels, from gene expression through cell proliferation dynamics to morphogenesis, have shown no traces of ancestral segmentation and thus, in line with palaeontological data, strongly suggest an unsegmented last common ancestral mollusc.
3. Polyplacophorans (as part of the aculiferan lineage) have retained a conserved, staggered Hox gene expression motif along the anterior–posterior body axis, while gastropods and cephalopods appear to deviate from this pattern and instead show Hox transcripts in lineage‐specific structures, suggesting that these key developmental regulators were recruited into the evolution of conchiferan morphological novelties.
4. The plasticity of molluscan developmental gene expression is further demonstrated by components of other axial‐patterning pathways such as the Dpp/BMP and the Nodal signalling cascades that likewise show considerable differences between molluscan subtaxa. Joining forces in a holistic framework where organismal knowledge is combined with large‐scale sequencing efforts and MorphoEvoDevo approaches will undoubtedly uncover many of the stories yet deeply hidden in molluscan evolutionary history.
